# Consumption of Stilbenes and Flavonoids is Linked to Reduced Risk of Obesity Independently of Fiber Intake

**DOI:** 10.3390/nu12061871

**Published:** 2020-06-23

**Authors:** Olatz Mompeo, Tim D. Spector, Marisa Matey Hernandez, Caroline Le Roy, Geoffrey Istas, Melanie Le Sayec, Massimo Mangino, Amy Jennings, Ana Rodriguez-Mateos, Ana M. Valdes, Cristina Menni

**Affiliations:** 1 Department of Twin Research & Genetic Epidemiology, King’s College London, St Thomas’ Hospital Campus, Westminster Bridge Road, London SE1 7EH, UK; olatz.mompeo@kcl.ac.uk (O.M.); mateyhernandezm@gmail.com (M.M.H.); caroline.le_roy@kcl.ac.uk (C.L.R.); massimo.mangino@kcl.ac.uk (M.M.); Ana.Valdes@nottingham.ac.uk (A.M.V.); 2 Department of Nutritional Sciences, King’s College London, Franklin-Wilkins Building, Stamford St, London SE1 9NH, UK; geoffrey.istas@gmail.com (G.I.); melanie.le_sayec@kcl.ac.uk (M.L.S.); ana.rodriguez-mateos@kcl.ac.uk (A.R.-M.); 3 NIHR Biomedical Research Centre at Guy’s and St Thomas’ Foundation Trust, London SE1 9RT, UK; 4 Norwich Medical School, University of East Anglia, Norwich NR4 7TJ, UK; amy.jennings@uea.ac.uk; 5 Academic Rheumatology, University of Nottingham, Hucknall Road, Nottingham NG5 1PB, UK

**Keywords:** polyphenol intake, obesity, gut microbiome diversity, gut microbiome composition, fiber intake, stilbene intake, flavonoid intake

## Abstract

Background: Polyphenol consumption is implicated in gut microbiome composition and improved metabolic outcomes, but it is unclear whether the effect is independent of dietary fiber. Methods: We investigated the links between (poly)phenol intake, gut microbiome composition (16s RNA) and obesity independently of fiber intake in UK women (*n* = 1810) and in a small group of UK men (*n* = 64). Results: (Poly)phenol intakes correlated with microbiome alpha diversity (Shannon Index) after adjusting for confounders and fiber intake. Moreover, flavonoid intake was significantly correlated with the abundance of *Veillonella*, (a genus known to improve physical performance), and stilbene intake with that of butyrate-producing bacteria (*Lachnospira* and *Faecalibacterium)*. Stilbene and flavonoid intake also correlated with lower odds of prevalent obesity (*Stilbenes:* Odds Ratio (95% Confidence Interval) (OR(95%CI)) = 0.80 (0.73, 0.87), *p* = 4.90 × 10^−7^; *Flavonoids*: OR(95%CI) = 0.77 (0.65, 0.91), *p* = 0.002). Formal mediation analyses revealed that gut microbiome mediates ~11% of the total effect of flavonoid and stilbene intake on prevalent obesity. Conclusions: Our findings highlight the importance of (poly)phenol consumption for optimal human health.

## 1. Introduction

Many of the gut microbiome’s effects on health parameters are the result of microbes metabolising diverse compounds from our diet [1]. Dietary (poly)phenols, which include flavonoids (such as flavan-3-ols, flavonols, anthocyanins, flavanones, flavones and isoflavones) and non-flavonoids (such as stilbenes, ellagitannins, lignans and phenolic acids), are actively studied for their potential health benefits in chronic diseases, such as cardiovascular diseases (CVD) and age-related cognitive decline [2]. Common (poly)phenol-rich foods include fruits, berries, nuts, tea, cocoa products, coffee, vegetables, olive oil, soy products and red wine [3].

Studies have shown that diets based on a high intake of plant (poly)phenols and fermentable fibers alter microbial activities within the gut [4]. Indeed, the gut microbiome converts (poly)phenols and fibers into biologically active compounds, thereby regulating energy and lipids metabolism [5] with potential impact on metabolic diseases [6]. (Poly)phenols, for instance, exert a protective effect on vascular endothelial cells by preventing the oxidation of low-density lipoprotein [7]. In animal studies, supplementation with high concentrations of polyphenols inhibits the growth of detrimental species, such as *Clostridia* and *Enterobacteria*, and increases the abundance of Lactic acid bacteria and *Akkermansia* [8].

Small interventional studies have shown that (poly)phenol consumption (e.g., from wine, cocoa, tea, blueberries or aronia berries) results in increases in specific beneficial bacterial communities in the gut, particularly *Bifidobacterium*, *Lactobacillus* and *Anaerostipes* [9,10,11,12,13], while the relative abundance of *Bacteroides* increases in subjects consuming red wine or aronia berry (poly)phenols [10,13,14]. A recent study from our group found that red wine consumption was associated with an increase in gut microbial alpha diversity, potentially mediating host body mass index (BMI) reduction in two cohorts [15]. (Poly)phenols have also been implicated in the reduced abundance of pathogenic bacterial species in the gut microbiome. A study examining the antibacterial activity of fruit (poly)phenols found a high sensitivity to these compounds in the enteropathogens *Staphylococcus aureus* and *Salmonella typhimurium* [16], while reductions in pathogenic *Clostridium* species (*C*. *perfringens* and *C. histolyticum*) have been reported following consumption of fruit, seed, wine and tea polyphenols [17,18,19,20].

(Poly)phenols are well known antioxidants [21], and the pivotal role of oxidative stress as a key pathway in weight gain has been highlighted before [22]. It has therefore been hypothesized that both flavonoids, such as curcumin, and stilbenes such as resveratrol, have beneficial effects on adipose tissue by alleviating intracellular oxidative stress, reducing chronic low-grade inflammation, inhibiting adipogenesis and lipogenesis, and suppressing the differentiation of preadipocytes to mature adipocytes [23].

However, it is not clear whether polyphenols and fermentable fibers act in tandem (or synergistically) through the gut microbiota to improve different physiological processes linked to metabolic disease risk, or whether they have independent effects [4].

Here we assess the association between (poly)phenol intake, as measured by food frequency questionnaires (FFQs), and gut microbial diversity and abundances, independently of fiber intake (measured as non-starch polysaccharides) in a large-population cohort. We then investigate whether the gut microbiome mediates part of the benefits of (poly)phenols intake on obesity. This can help prioritize interventions.

## 2. Methods

### 2.1. Study Populations

Discovery cohort: The Discovery cohort included female twins enrolled in the TwinsUK registry, a national register of adult twins recruited as volunteers without selecting for any particular disease or traits [24]. Here we analyzed data from 1810 female twins aged 62.03 (Standard Deviation (SD) = 11.58), 71.4% of them post-menopausal, with 16s microbiome data and (poly)phenol intake measured within 1.59 (SD = 1.08) years (Table 1). A subset of 508 study participants also had concurrent fecal lactate measured by mass spec (Metabolon inc) [25]. The TwinsUK study was approved by St Thomas’ Hospital Research Ethics Committee, and all twins provided written informed consent.

Validation cohort: We also included a small validation cohort, the Aronia study, of 64 helthy male volunteers aged 18–45 y, recruited from King’s College London and the nearby areas [13] (Table 1). This replication cohort was chosen given the very different pattern from polyphenol cohort, to enable assessing the generalizability of associations between gut microbiome and (poly)phenol intake. The study was a double blind randomized controlled trial, with the main aim of investigating the effects of aronia berries on vascular function and gut microbiota composition in a healthy population. The study was approved by King’s College London Ethics Committee (HR-15/16–3739) and the trial was registered at clinicaltrials.gov as NCT03041961. The main findings of this study have been previously published [13]. For the purposes of this work, baseline clinical characteristics, dietary data on (poly)phenol and fiber intake, and 16s microbiome data are presented here.

(Poly)phenols and fiber intake: A validated 131-item semi-quantitative FFQ established for the European Prospective Investigations into Cancer and Nutrition (EPIC)-Norfolk study [26] was used to assess dietary intake in both the TwinsUK and the Aronia study. Estimated intakes of fiber (non-starch polysaccharides, in grams per day) were derived from the UK Nutrient Database [27] and were adjusted for energy intake using the residual method prior to analysis [28]. Estimated intakes of total (poly)phenols (in mg per day) were derived from the Phenol-Explorer Database [29]. The main sources of (poly)phenol intake are reported in Table S1.

Obesity: Measurements of weight and height were used to calculate body mass index (BMI), calculated as weight in kilograms divided by the square of height in meters. Participants were classified as obese if their BMI was greater than 30.

### 2.2. Microbiota Analysis

Gut microbiome composition was determined by sequencing of the 16s rRNA gene, as previously described [30]. Fecal samples were brought to clinical visit or posted in sealed ice packs and frozen at −80 °C, before being sent on dry ice to Cornell University where DNA was amplified. Amplicons were sequenced on an Illumina MiSeq platform and samples read. Paired ends were merged following demultiplexing. Operational taxonomic units(OTUs) were generated from the 16s rRNA gene sequencing and collapsed under taxonomic units at the genus level as previously described [31]. Residuals for genera were generated via regression against technical covariates including sequencing depth, sequencing run, sequencing technician and sample collection method. These residuals were inverse normalized, as they were not normally distributed, and used in downstream analyses. In order to calculate alpha diversity, the complete OTU count table was rarefied to 10,000 sequences per sample 50 times. Alpha diversity metrics were calculated for each sample in each of the rarefied tables, and final diversity measures taken as the mean score across all 50. Alpha diversities were quantified as observed OTU counts and Shannon diversity index. Alpha diversity indexes were standardized to have mean 0 and SD 1.

Regarding fecal samples from the Aronia study, gut microbiota composition was also determined via 16s RNA sequencing as previously described [13].

### 2.3. Availability of Data and Materials

16s sequencing data used for this study is deposited in the European Nucleotide Archive (ERP015317). All other TwinsUK data are available upon request on the department website. 

### 2.4. Statistical Analysis

Statistical analysis was carried out using R studio version 3.5.1. We normalized measures of (poly)phenol intake by taking the log. We assessed the association between alpha diversity indexes (outcome) and measures of (poly)phenol intake (exposure) by using a linear mixed model with family as a random intercept, adjusting for age, BMI, dietary fiber and daily energy intake. Linear/logistic mixed models, adjusting for age, BMI, dietary fiber and daily energy intake, and family relatedness were also employed to investigate the association between (i) taxa (outcome: genus with an abundance > 0.001) and (poly)phenol intake (stilbenes/flavonoids—exposure), adjusting for multiple testing using false discovery rate (FDR < 0.05); and (ii) obesity and (poly)phenol (stilbenes/flavonoids—exposure) intake, adjusting for the aforementioned variables but BMI.

We further employed mediation analysis as implemented in the R package “mediation” [32] to test the mediation effects of Shannon diversity (indirect effect) on the total effect of (poly)phenol intake (stilbene/flavonoid) on obesity (BMI > 30 kg/m^2^), adjusting for age, fiber, daily energy intake and family structure. We constructed a mediation model to quantify both the direct effect of (poly)phenols intake on obesity, and the indirect (mediated) effects mentioned above. The variance accounted for (VAF) score, which represents the ratio of indirect-to-total effect and determines the proportion of the variance explained by the mediation process, was further used to determine the significance of mediation effect [33].

## 3. Results

The demographic characteristics of the study population are presented in Table 1.

Correlations between dietary (poly)phenol intake and gut microbiome diversity and composition.

**Figure 1:**
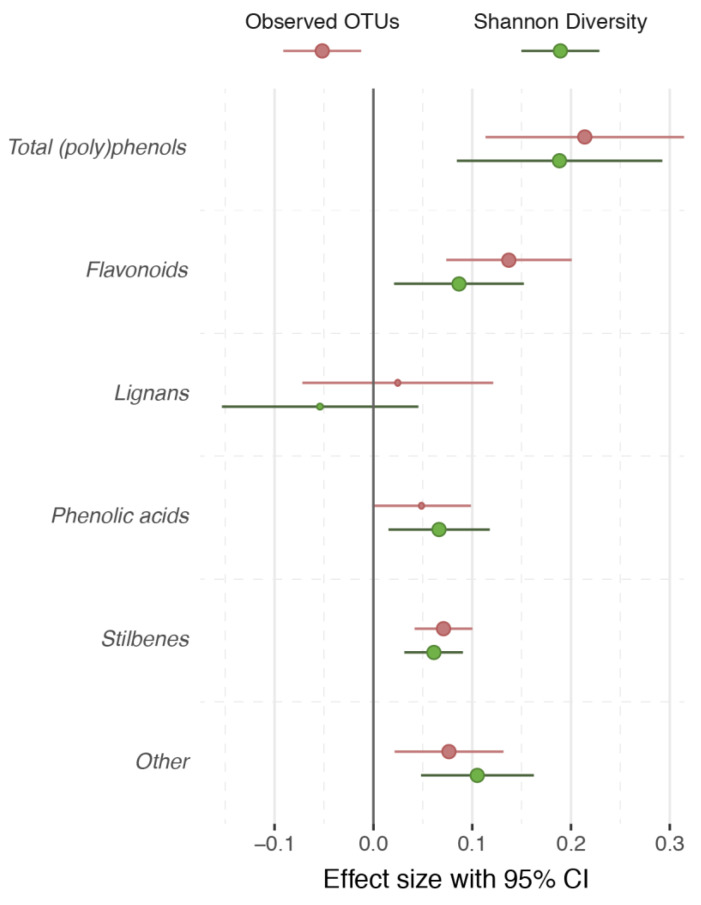
Dietary (poly)phenols and measures of gut microbiome alpha diversity in the TwinsUK cohort.

In 1810 female twins from the TwinsUK cohort, with microbiome data available and (poly)phenol intake from FFQs measured within 1.59 years (SD = 1.08), we find that both Shannon diversity and the number of observed OTUs were significantly associated with total (poly)phenol intake (*Shannon*: Beta (SE) = 0.19 (0.05), *p* = 3.77 × 10^−4^, *Observed number of OTUs*: 0.21 (0.05), *p* = 2.99 × 10^−5^), stilbene (*Shannon*: 0.06 (0.02), *p* = 5.42 × 10^−5^, *Observed number of OTUs*: 0.07 (0.01), *p* = 6.51 × 10^−3^) and flavonoid intake (*Shannon*: 0.09 (0.03), *p* = 9.67 × 10^−3^, *Observed number of OTUs*: 0.14 (0.03), *p* = 2.31 × 10^−5^) after adjusting for age, BMI, fiber intake, energy intake and family relatedness (Figure 1). We validated the association between Shannon diversity and total (poly)phenol intake, adjusting for fiber intake, age and BMI, in a small independent cohort of 64 men from the Aronia study [13] (Beta (SE) = 0.25 (0.11), *p* = 0.02).

Analyses were adjusted by age, BMI, dietary fiber intake, total energy intake and family relatedness. CI indicates confidence interval.

**Figure 2:**
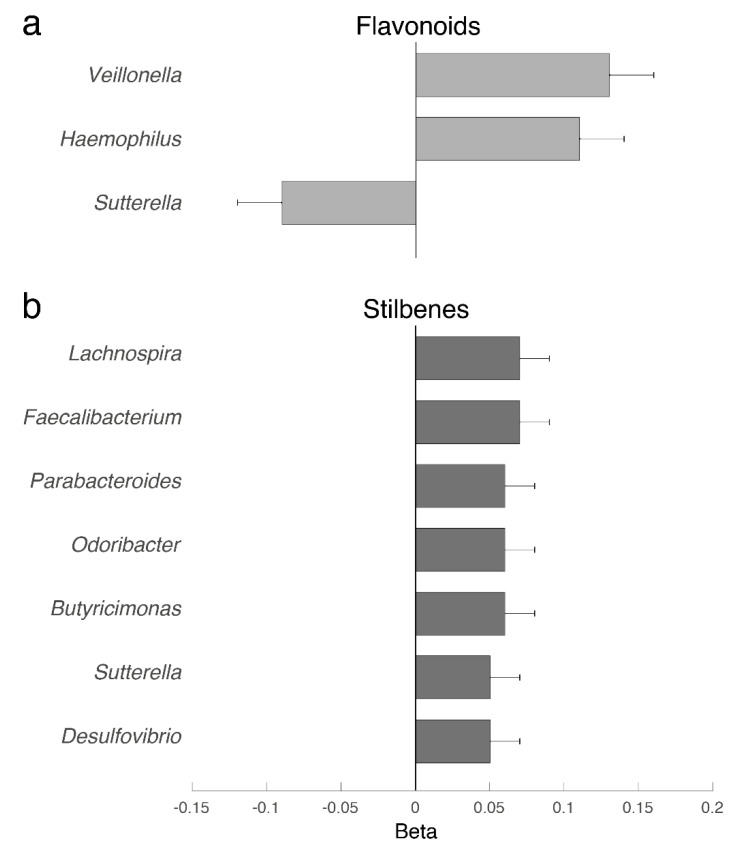
(**a**) Genus–flavonoid intake association. (**b**) Genus–stilbene intake association. Analyses are adjusted for age, BMI, fiber intake, energy intake and multiple testing (FDR < 0.05).

We then examined, in the TwinsUK cohort, the association between stilbene and flavonoid intakes and bacterial abundances (genus with abundance > 0.001). We identified five genera significantly associated with stilbene intake and three genera associated with flavonoid intake, after adjusting for age, BMI, fiber intake, energy intake, family relatedness and multiple testing using FDR correction (FDR < 0.05) (Figure 2). These include (i) the positive correlation of flavonoid intake and *Veillonella*, a performance-enhancing microbe that functions via its metabolic conversion of exercise-induced lactate into the short-chain fatty acid propionate [34], and (ii) the positive correlation of stilbene intake with some known butyrate-producing bacteria, such as *Lachnospira* and *Faecalibacterium*.

In addition, we found that *Veillonella* abundances were positively correlated with fecal abundance of lactate (Beta (SE) = 0.27 (0.04), *p* = 1.45 × 10^−10^) in TwinsUK.

Correlations between dietary stilbene and flavonoid intakes, obesity and gut microbiome diversity.

We next investigated, in the TwinsUK cohort, the associations between stilbene and flavonoid intake and obesity, adjusting for covariates, fiber intake and family structure. We found that both stilbene and flavonoid intake (in the log scale) were also associated with a significantly lower prevalence of obesity. (*Stilbenes*: OR(95%CI) = 0.80 (0.73,0.87), *p* = 4.90 × 10^−7^; *Flavonoids*: OR(95%CI) = 0.77 (0.65,0.91), *p* = 0.002) (Table 2).

Analyses are adjusted for age, fiber intake, total daily energy intake and family relatedness.

We therefore conducted a formal mediation analysis with TwinsUK to determine the indirect effect of the gut microbiome (Shannon index) on the relationship between (poly)phenol intake (stilbene- and flavonoid-high versus -low) and obesity. The analysis found that the Shannon diversity acted as a potential partial mediator in the negative association between stilbene intake and obesity (VAF = 11.16% (8.13%, 16.66%) *p* < 0.001), and in the negative association between flavonoids intake and obesity (11.11% (4.71%, 93.80%)).

## 4. Discussion

In the largest study to date, we report that (poly)phenol intake, and particularly stilbenes and flavonoids, are associated with higher gut microbiota diversity and lower prevalence of obesity, independent of fiber intake. Furthermore, gut microbial diversity mediated the association between stilbene and flavonoid intakes and obesity. In addition, we identified a positive correlation between flavonoid intake and *Veillonella,* the energy-enhancing bacteria, and a positive association between stilbene intake and *Faecalibacterium,* a key marker of a healthy gut.

Stilbenes are non-flavonoid (poly)phenols, characterized by the presence of a 1,2-diphenylethylene nucleus in their structure, and include resveratrol and its derivatives [35]. They are present in different foods and plants at high levels, such as red grapes, red wine, some kinds of tea, berries and peanuts [36], though their levels are very low in foods overall. Because of this, the association between gut microbiome diversity and stilbene intake may be a marker of other factors, such as consumption of red wine and berries, which are also very rich in other (poly)phenols, such as anthocyanins and flavan-3-ols. However, when we repeated the analyses adjusting for anthocyanins and flavan-3-ols, the results remained the same, suggesting that the effect of stilbenes is independent.

Several small studies have found that polyphenols might be effective in preventing small increases in weight during periods of overfeeding, although there is no evidence for them reducing weight [37]. Our finding of a 20–23% lower prevalence of obesity with higher intakes of total flavonoids and stilbenes, of which up to 11% is mediated by microbial diversity, highlights the public health importance of these results. By increasing the dietary intake of polyphenols, it may be possible to prevent some of the weight gain which is constantly increasing worldwide [38].

The intakes associated with these findings could be readily incorporated into the diet by encouraging a Mediterranean diet, which facilitates a higher polyphenol intake than the traditional western diet [39], or by including plants such as Hibiscus [21] as part of the diet.

The flavonoid class embraces the largest and most widely studied group of (poly)phenols, with a structure characterized by two phenyl rings and a heterocyclic ring. The main food sources of flavonoids in the TwinsUK cohort are tea, dark chocolate, cocoa and fruit (Table S1). Previous studies have suggested that (poly)phenols and fiber may provide complementary health benefits [40]. Only a small percentage (<10%) of the (poly)phenols ingested is absorbed in the small intestine. The rest accumulates in the lumen, and is subjected to the catabolic reactions of the gut microbial community, which breaks them down into a series of low molecular weight phenolic metabolites that, being absorbable, may be responsible for the health benefits of (poly)phenols [41].

According to current UK dietary guidelines, the recommended average fiber intake for adults is 30g per day [42]. However, though the dietary guidelines suggest that adults should consume five portions of fruits and vegetables per day [43], and though the newly published Cardiovascular disease risk Reduction (CRESSIDA) study shows that adherence to UK dietary guidelines is associated with higher dietary intake of total and specific (poly)phenols [44], there is no indication as to the amount of total and specific (poly)phenols that should be ingested. We found that (poly)phenols, and in particular stilbenes and flavonoids, are associated with microbiome diversity independently of fiber intake. Our results in both the TwinsUK and Aronia cohorts indicate that even moderate consumption of (poly)phenols in the diet may have a positive impact on gut microbiome diversity. As the range of (poly)phenols in fruits and vegetables is highly variable [45], our results could inform new guidelines for increasing awareness of the beneficial effects of (poly)phenol consumption. More importantly, our study highlights that different types of phenolic compounds have different links to gut microbiome composition, which may in turn have different effects on human health. Future studies are needed to look at more specific sub-classes of polyphenols, and the food sources of these polyphenols, as they will have structurally diverse polyphenol profiles, which is likely to lead to differing interactions with the gut microbiome.

In our exploratory study, we also report a link between flavonoid dietary intake and the abundance of *Veillonella*. A recent study by Schelman and coworkers [34] found that not only is the abundance of this bacterium linked to improved exercise performance, but that this is due to a transformation of lactate into propionate. Moreover, in our data, we find a significant positive association between *Veillonella* and the fecal abundance of lactate. The association between *Veillonella* and flavonoid intake then suggests one of the mechanisms whereby flavonoids may improve metabolic health, as up to certain doses, propionate is expected to improve energy metabolism. It also suggests that there may be scope for investigating the role of flavonoid supplementation or dietary interventions in improving athletic performance.

Another interesting aspect to come out of our study is the higher abundance of *Faecalibacterium* in individuals with higher stilbene intakes. *Faecalibacterium prausnitzii* is known to be a major butyrate producer [46], and it has been widely claimed that among the main benefits of prebiotic fibers, such as inulin, is the increase in butyrate production [47], a powerful anti-inflammatory and histone deacetylase regulator [48] shown to be protective against colon cancer [49]. This suggests that some of the health benefits commonly associated with stilbenes, such as resveratrol, may be due to their link to butyrate production, and this is an aspect that deserves further investigation.

We note some study limitations. First, we have used FFQs rather than other methods for assessing nutrient intake. FFQs have become a well-accepted method for the quantitative assessment of usual nutrient intake [50], but being recalled, the data are subject to some bias. However, the value of FFQs for assessing dietary composition has been documented objectively, by correlations with biochemical indicators and the prediction of outcomes in prospective studies [51,52]. Second, the discovery cohort was based on middle-aged white female twins. However, we replicated our main results in a small independent study of males, suggesting that the results are generalizable to both sexes. Thirdly, the cross-sectional and observational nature of this study does not allow us to determine causal relationships between (poly)phenol intake, gut microbiome composition and health, where ideally randomized control trials are needed. It does not allow us to understand some associations, which go in the opposite direction for flavonoids and stilbenes, such as the one with *Sutterellla*. Moreover, we here investigated the distinct impact of polyphenols on the microbiome using only bio-informatics tools. Additional investigations, involving diets with omitted polyphenols or dietary fibers, are recommended to validate the current findings. Further, an important future avenue for research will be investigating whether any of these interactions between (poly)phenols and gut microbes are different in men vs women, or young vs old, and if the type and amount of polyphenol intake can be optimized based on demographic considerations. On the other hand, we note several strengths, including the sample size of the study and the detailed clinical and molecular phenotyping of the study subjects, which has allowed us to test the relative contributions of (poly)phenol intake to metabolic phenotypes.

## 5. Conclusions

In conclusion, stilbene, flavonoid and total (poly)phenol intake was associated with higher gut microbiome diversity independently of fiber intake, suggesting that polyphenols provide complementary health benefits to fiber, and may be included in an expanded prebiotics group. Our results also suggest that gut microbiome diversity mediates the reverse association of (poly)phenols with obesity, increasing our understanding of how they interact with the gut microbiota. Our results indicate that the beneficial impact of (poly)phenol consumption on metabolic health may be partially driven by the interaction between the type of phenolic compound in the diet and the gut microbiota. Additional studies, including dietary intervention studies, exploring these associations in relation to health and diseases, are required to provide a sustainable basis for dietary recommendations.

## Figures and Tables

**Table 1:** Demographic characteristics of the study population.

Phenotype	TwinsUK	Aronia
*n*	1810	64
Females, *n* (%)	1810 (100%)	0
Post-Menopausal, *n* (%)	1287 (71.4%)	0
Hypertension, *n* (%)	601 (49.71%)	0
Obesity, *n* (%)	320 (17.68%)	0
Type 2 diabetes, *n* (%)	73 (4.33%)	0
	*Mean (SD)*	
Age, yrs	61.31 (11.27)	23.61 (0.59)
BMI, kg/m^2^	25.83 (4.77)	22.77 (2.12)
Energy intake, kcal	1806.88 (527.45)	
Fiber intake, g/day	19.88 (7.26)	14.33 (6.94)
(Poly)phenol intake, mg/day		
Total (poly)phenol intake	1488.36 (585.19)	432.40 (421.55)
Flavonoids	724.68 (385.46)	NA
Lignans	85.70 (49.89)	NA
Phenolic acids	637.80 (491.36)	NA
Stilbenes	0.81 (0.96)	NA
Other (poly)phenols	39.37 (31.51)	NA
*Measure of alpha diversity*		
Shannon Diversity	5.16 (0.72)	2.61 (0.59)
Observed number OTUs	348.10 (102.23)	NA

**Table 2:** Obesity–(poly)phenol intake (stilbenes and flavonoids) association.

	Stilbenes			Flavonoids		
	OR	SE	*P*	OR	SE	*P*
**Obesity**	0.80	0.04	4.90 × 10^−7^	0.77	0.07	0.002

## Supplementary Materials

The following are available online at https://www.mdpi.com/2072-6643/12/6/1871/s1, Table S1: Estimated average (poly)phenol intake from food groups in TwinsUK, *mean(SD)* in mg/100/day.
