# Supplementary material for: Consumption of Stilbenes and Flavonoids is Linked to Reduced Risk of Obesity Independently of Fiber Intake
Source: Nutrients. 2020 Jun 23;12(6):1871. doi: 10.3390/nu12061871 (PMC7353284; doi:10.3390/nu12061871)
Supplement: Supplementary file 1 [file nutrients-12-01871-s001.zip › Supplementary material.docx]

**Table S1. Estimated average (poly)phenol intake from food groups in TwinsUK, *mean(SD)* in mg/100/day**

| Food Groups | Total (poly)phenols | Flavonoids | Stilbenes |
| --- | --- | --- | --- |
| Baked beans | 0.76(0.9) | 0.01(0.02) | 0(0) |
| Beer | 0.49(1.8) | 0.1(0.37) | 0(0) |
| Berries | 19.19(24.29) | 14.27(18.06) | 0.02(0.02) |
| Butter | 0.03(0.1) | 0(0) | 0(0) |
| Chocolate Bars And Jam | 24.07(52.13) | 23.74(51.42) | 0(0) |
| Citrus fruit | 12.72(17.02) | 9.55(13.04) | 0(0) |
| Cocoa, Coffee and Fruit Tea | 203.67(393.14) | 49.86(156.47) | 0(0) |
| Coffee | 356.79(412.89) | 0(0) | 0(0) |
| Cooked potatoes | 10.16(8.77) | 0(0) | 0(0) |
| Crisp bread | 2.96(7.36) | 0(0) | 0(0) |
| Dairy products highfat | 10.25(13.35) | 8.95(11.66) | 0.02(0.02) |
| Dairy products lowfat | 4.36(8.24) | 3.98(7.27) | 0(0.01) |
| Fried potatoes | 3.41(3.18) | 0(0.02) | 0(0) |
| Fruit | 108.31(92.44) | 89.08(79.6) | 0.05(0.07) |
| Fruit juice | 24.97(44.52) | 21.89(39.19) | 0.01(0.03) |
| High fibre breakfast cereals | 17.06(30.68) | 0.95(1.83) | 0(0) |
| Lasagne | 0.36(0.47) | 0.04(0.06) | 0(0) |
| Legumes | 7.35(6.5) | 1.69(1.44) | 0.01(0.01) |
| Low fibre breakfast cereals | 0(0) | 0(0) | 0(0) |
| Low fat spread | 0.01(0.07) | 0(0) | 0(0) |
| Margarine | 0.11(0.23) | 0(0) | 0(0) |
| Nuts | 13.47(25.14) | 1.16(2.38) | 0(0) |
| Pizza | 0.59(0.79) | 0.25(0.33) | 0(0) |
| Porridge | 0(0) | 0(0) | 0(0) |
| Pufa margarine | 0.06(0.16) | 0(0) | 0(0) |
| Savoury pies | 0.19(0.31) | 0.08(0.14) | 0(0) |
| Savoury snacks | 0.69(0.94) | 0.08(0.18) | 0(0) |
| Soy foods | 0.22(0.98) | 0.2(0.89) | 0(0) |
| Spirits and liquor | 0.03(0.16) | 0.01(0.07) | 0(0) |
| Sweet baked | 5.21(7.17) | 4.18(6.38) | 0(0) |
| Tea | 519.07(390.66) | 443.13(333.51) | 0(0) |
| Vegetables allium | 1.14(0.99) | 0.68(0.57) | 0(0) |
| Vegetables cruciferous | 3.26(3.27) | 0.53(0.76) | 0(0) |
| Vegetables greenleafy | 54.19(44.56) | 0.78(0.68) | 0(0) |
| Vegetables other | 6.85(4.68) | 0.09(0.08) | 0(0) |
| Vegetables yellow | 3.57(4.2) | 2.48(3.82) | 0(0) |
| White and brown bread refined grains | 5.33(5.21) | 0.11(0.2) | 0(0) |
| Whole meal bread and grains | 9.19(11.18) | 0(0) | 0(0) |
| Wine | 19.44(27.51) | 13.46(20.35) | 0.71(0.96) |
